# Feeding Formula Eliminates the Necessity of Bacterial Dysbiosis and Induces Inflammation and Injury in the Paneth Cell Disruption Murine NEC Model in an Osmolality-Dependent Manner

**DOI:** 10.3390/nu12040900

**Published:** 2020-03-26

**Authors:** Shiloh R Lueschow, Stacy L Kern, Huiyu Gong, Justin L Grobe, Jeffrey L Segar, Susan J Carlson, Steven J McElroy

**Affiliations:** 1 Department of Microbiology and Immunology, University of Iowa, Iowa City, IA 52242, USA; shiloh-lueschow@uiowa.edu; 2 Stead Family Department of Pediatrics, University of Iowa, Iowa City, IA 52242, USA; denn0122@umn.edu (S.L.K.); huiyu-gong@uiowa.edu (H.G.); susan-carlson@uiowa.edu (S.J.C.); 3 Department of Physiology, Medical Coll5ge of Wisconsin, Milwaukee, WI 53226, USA; jgrobe@mcw.edu; 4 Department of Pediatrics, Medical College of Wisconsin, Milwaukee, WI 53226, USA; jsegar@mcw.edu

**Keywords:** necrotizing enterocolitis, immature intestine, formula, osmolality, inflammation, microbiome

## Abstract

Necrotizing enterocolitis (NEC) remains a significant cause of morbidity and mortality in preterm infants. Formula feeding is a risk factor for NEC and osmolality, which is increased by the fortification that is required for adequate growth of the infant, has been suggested as a potential cause. Our laboratory has shown that Paneth cell disruption followed by induction of dysbiosis can induce NEC-like pathology in the absence of feeds. We hypothesized adding formula feeds to the model would exacerbate intestinal injury and inflammation in an osmolality-dependent manner. NEC-like injury was induced in 14–16 day-old C57Bl/6J mice by Paneth cell disruption with dithizone or diphtheria toxin, followed by feeding rodent milk substitute with varying osmolality (250–1491 mOsm/kg H_2_O). Animal weight, serum cytokines and osmolality, small intestinal injury, and cecal microbial composition were quantified. Paneth cell-disrupted mice fed formula had significant NEC scores compared to controls and no longer required induction of bacterial dysbiosis. Significant increases in serum inflammatory markers, small intestinal damage, and overall mortality were osmolality-dependent and not related to microbial changes. Overall, formula feeding in combination with Paneth cell disruption induced NEC-like injury in an osmolality-dependent manner, emphasizing the importance of vigilance in designing preterm infant feeds.

## 1. Introduction

Necrotizing enterocolitis (NEC) remains the leading cause of gastrointestinal morbidity and mortality of premature infants, leading to the death of 1/3 to ½ of the infants who develop disease [1,2]. Although the pathophysiology of NEC is still unknown, it is postulated to be the result of bacterial translocation across the immature epithelial barrier leading to tissue invasion, subsequent inflammation, and ultimately destruction [3,4]. In general, NEC onset is preceded by a bloom in Enterobacteriaceae species, although there has been no specific microorganism determined to be causative [5,6,7,8,9,10,11]. Studies have linked bacterial dysbiosis or microbiome disruption with NEC development and many murine NEC models capitalize on this fact by inducing bacterial dysbiosis, disruption of the microbiome, or utilizing lipopolysaccharide (LPS), particularly in young mice [12,13,14,15,16]. Formula feeding has been associated with increased risk for development of NEC [17] and in rat models has been shown to induce an inflammatory response in the immature intestine [18]. As an inflammatory milieu can induce dysbiosis in the immature intestine [6], it is reasonable to think that formula-induced inflammation may be one cause of the dysbiosis seen prior to development of NEC.

Our prior work has shown that NEC-like injury can be induced in 14–16 day-old mice (intestinally equivalent to a 22–24 week infant [19,20]) by disrupting Paneth cell biology followed by creation of an intestinal bacterial dysbiosis [21,22,23,24]. This model has direct relevance to human biology as multiple investigators have found a decrease in Paneth cells or their granular components following development of NEC [25,26,27,28] and an association with NEC and genetic disturbances in Paneth cell biology [29]. We mimic this Paneth cell disruption in wild type mice with dithizone treatment, or in genetically susceptible mice by exposure to diphtheria toxin [21,22,23,24,30,31]. Dithizone is a heavy metal chelator that disrupts zinc-rich Paneth cells by leading to an upregulation in autophagy pathways [31]. In contrast, our *PC-DTR* strain of mice has a human diphtheria toxin receptor bound to the Paneth cell-specific cryptdin 2 promoter [30,31,32], and exposure of these mice to diphtheria toxin induces Paneth cell-specific apoptosis. Following either method of Paneth cell disruption, enteral exposure of *Klebsiella pneumoniae* at a concentration of 1 × 10^11^ colony forming units (CFU)/kilogram body weight to induce bacterial dysbiosis leads to consistent and rapid intestinal pathology that mimics human NEC [26]. Although this model represents two risk factors for NEC (intestinal immaturity and microbial dysbiosis) injury is induced while the mice are not fed.

Infants fed formula are at significantly more risk for NEC than those fed human milk [17,33,34,35]; however, the mechanism behind this discrepancy is unknown. Human breast milk contains many unique components including nutrients, immunoglobulins, growth factors, hormones, sugars, and proteins that nurture and help to immunologically protect the developing infant. In this fashion, human breast milk is tailored to feed term infants and has everything needed for these infants to grow and succeed [36,37]. However, the caloric requirements necessary to maintain the fetal growth trajectory after birth in preterm infants cannot be achieved by breast milk alone and thus requires fortification from an external source [38,39]. Providing adequate nutrition to preterm infants is critical as there is considerable evidence to support a relationship between poor post-natal growth and later development of neuro-developmental sequelae [40,41,42].

One side effect of breast milk fortification is a significant increase in osmolality [43]. Osmolality is a measure of the osmoles of solute per kilogram of solvent, with osmoles being defined as a unit of osmotic pressure equivalent to the amount of solute that dissociates in solution to form one mole of particle [44]. The osmolality of infant formula is important as it has been suggested that ingestion of hyperosmolar feeds may lead to NEC [45,46,47]. While this suggestion was first proposed almost 50 years ago, the understanding of the effect that osmolality has on the immature intestine remains limited. Two recent studies have shown that formula feeding induces changes in intestinal microcirculation and that the associated injury is not osmolality-dependent [48,49]. However, these studies used five- to nine-day-old mice which have not yet developed Paneth cells [20,50] and thus could represent a more immature intestinal epithelium than what is seen in infants who are most susceptible to developing NEC [2,51,52]. To help clarify these gaps in knowledge, we set out to examine the effect of adding formula feeding to our NEC model and to understand if formula induced injury was osmolality dependent. Our novel data show that the addition of formula feeding to either dithizone- or diphtheria toxin-induced Paneth cell disruption eliminated the need to induce dysbiosis to cause NEC-like injury. We additionally show that intestinal injury and markers of inflammation were osmolality-dependent, but not necessarily microbiome dependent. Furthermore, the effects observed were dependent on the method of increasing osmolality. These data expand our understanding of how formula feeding may increase the risk of developing NEC in the preterm infant.

## 2. Methods

### 2.1. Animals and Feeding Protocols 

C57BL/6 mice were bred at the University of Iowa under standard conditions according to protocols approved by the Institutional Animal Care and Use Committee (Protocol #8041401) which was approved on 06/11/2018. Original founders were purchased from Jackson Laboratories (Bar Harbor, ME, USA). All mice were dam fed prior to experiments and, unless otherwise indicated, experiments were conducted with P14–P16 mice. All animals were dam fed prior to experimentation. On the day of experimentation, animals were separated from their mothers and maintained in a temperature and humidity-controlled chamber for the duration of the experiment. Formula feeds [23,53] were prepared and homogenized as previously described from their elemental components. Osmolality was measured using an OsmoPro Multi-Sample Micro-Osmometer (Advanced Instruments, Norwood, MA, USA). High osmolality formulas were achieved by adding Mannitol (MAN; Sigma-Aldrich, St. Louis, MO, USA), or by tripling the salt concentration of rodent milk substitution formula (RMS). Sham animals were given equal volumes of saline (Sigma-Aldrich, St. Louis, MO, USA). All prepared formulas and feeding solutions were given via a 24 × 1 W/1-1/4 blunt animal feeding needle (Cadence, Inc.) and a BD one ml Tuberculin syringe (Becton, Dickinson and Company, Franklin Lakes, NJ, USA). Mice were gavage fed 250 µL every three hours × four feeds with one of the following formulations: saline alone; RMS, MAN in saline, NaCl in saline (SALT), RMS^MAN^ added, or RMS^SALT^ (Table 1). Mice were weighed prior to the first feed and following the last feed. Mice were monitored for 3 hours after the final feed and were then euthanized for tissue harvesting. All experiments were performed on at least two separate occasions using at least two different litters. All controls were littermates from experimental animals.

### 2.2. Formula NEC Models

NEC was induced in postnatal day P14–P16 C57Bl/6J mice by giving an intraperitoneal injection of 75 mg/kg body weight dithizone (Sigma) dissolved in 25 mM LiCO_3_, or an equivalent volume of LiCO_3_ buffer alone. Depending on the group being considered, 250 µL of RMS (regular formula NEC), RMS^SALT^ (high osmolality formula NEC), or saline (control animals) was fed through oral gavage one hour prior to injecting with dithizone and every three hours for a total of four feeds. Mice were sacrificed and tissues were harvested thirteen hours after the first RMS/saline gavage. NEC was induced in P14–P16 *PC-DTR* mice by giving an intraperitoneal injection of diphtheria toxin at a concentration of 40 ng/g body weight and oral gavage of saline (control animals) or RMS (formula NEC) one hour prior to injection as well as every three hours for a total of four feeds. Mice were sacrificed at sixteen hours after the first oral gavage and tissues were harvested. Similar to above, all experiments were performed on at least two separate occasions using at least two different litters and all controls were littermates from experimental animals.

### 2.3. Serum and Cytokine Analysis

Blood was obtained from the facial vein at the time of euthanasia and serum was processed as previously described [24,54]. Serum samples were used for cytokine (KC-GRO (mouse ortholog to IL-8), TNF-α, IL-6, IL-1β, and IL-10) quantification using a Meso-Scale Discovery V-Plex assay (Meso-Scale, Gaithersburg, MD) on a Mesoscale Sector Imager 2400. Serum osmolality was quantified using an OsmoPro Multi-Sample Micro-Osmometer (Advanced Instruments, Norwood, MA, USA).

### 2.4. Microbiota Analysis

Mice were sacrificed according to institutional guidelines at the University of Iowa and ceca were harvested for DNA extraction using the Zymo Fecal/Soil Microbe MiniPrep kit (Zymo Research, Irvine, CA, USA) as previously described [55,56]. Amplification and sequencing were performed on the 16s rRNA V4 domain using a barcoded forward primer 515F (5′-NNNNNNNNGTGTGCCAFCMGCCGCCGCGGTAA-3′) and the reverse primer R806 (5′-GGACTACHVGGGTWTCTAAT-3′). The master mix for amplification of the DNA was made with 1x GoTaq Green Master Mix (Promega, Madison, WI, USA); 1 mM MgCl2; 200 nM 806R reverse primer; 200 nM 515F forward primer with barcode; nuclease free H20; and DNA. The thermocycler parameters for the PCR reaction were as follows: 94 °C for three minutes; 25 cycles of 94 °C for 45 s, 50 °C for 60 s, and 72 °C for 90 s, followed by 72 °C for 10 min and a 4 °C hold period indefinitely. PCR amplicons were run via gel electrophoresis to confirm amplification and then were pooled to relatively equal amplification intensities. The pooled amplicons were then resuspended in 2X the volume of Binding Buffer NTI from the Nucleospin PCR and Gel Purification Kit (Macherey-Nagel, Düren, Germany). The Nucleospin PCR Purification protocol was followed according to the manufacturer instructions (Macherey-Nagel, Düren, Germany). The purified pools were then quantitated using QuantIT dsDNA High Sensitivity Assay (Invitrogen, Carlsbad, CA, USA). Libraries were then submitted to the UC Davis DNA Technologies Center for 2 × 300 paired end Illumina MiSeq sequencing. Sequence data was analyzed using QIIME 1 (University of Colorado, Boulder, CO, USA, version 1.9.1) [57]. Sequences were first quality filtered and demultiplexed. Then, UCLUST (drive5.com, Tiburon, CA, USA) was used to assign operational taxonomic units (OTUs) based on a 97% pairwise identity. The OTUs went through a secondary filtration for low abundance OTUs at a 0.005% threshold. After both filtrations, the Ribosomal Database Project database (Michigan State University, East Lansing, MI, USA) was used to taxonomically classify the OTUs and compare with a representative set of the Greengenes 16s rRNA database (Second Genome, South San Francisco, CA, USA, gg_13_5 release). PyNAST (University of Colorado, Boulder, CO, USA) was used to align the OTU sequences and construct a phylogenetic tree for further β diversity analysis. Unweighted and abundance weighted UniFrac distance was used to calculate the β diversity. Samples were clustered based according to distances between samples.

### 2.5. Injury Scoring

Ileal samples were stained with hematoxylin and eosin to determine injury scores. For NEC models, injury was scored based on a standardized scale as we have described previously [22,23,24,31] from zero (healthy intestine) to four (full thickness necrosis). Scores of two or greater (separation from the basement membrane and disruption of the villus core) were considered significant for NEC. General non-NEC intestinal injury was assessed by two separate blinded investigators on a three-point scale evaluating villus integrity and separation from the basement membrane as previously described [55,58]. A score of zero was used to describe normal mucosa. Mild injury (score of one) encompassed the development of subepithelial Gruenhagen’s space, vacuolization or subepithelial lifting limited to the lamina propria or tips of villi. Severe injury (score of two) involved epithelial lifting and vacuolization greater than half of the villi, villi distortion, or mucosal ulceration and disintegration of the lamina propria.

### 2.6. Statistical Analysis

All experiments were performed in at least triplicate and all experiments used animals from at least 2 separate litters. Specific sample sizes are denoted in the Figure legends. ANOVA and non-parametric Kruskal-Wallis testing was performed to determine statistical significance using Graph Pad Prism v8. Significance was set as *p* < 0.05 for all experiments.

## 3. Results

### 3.1. Formula Feeding in Combination with Dithizone Treatment Results in Inflammation and Removes the Requirement for Bacterial Exposure to Induce NEC-Like Injury in the Immature Murine Intestine

**Figure 1:**
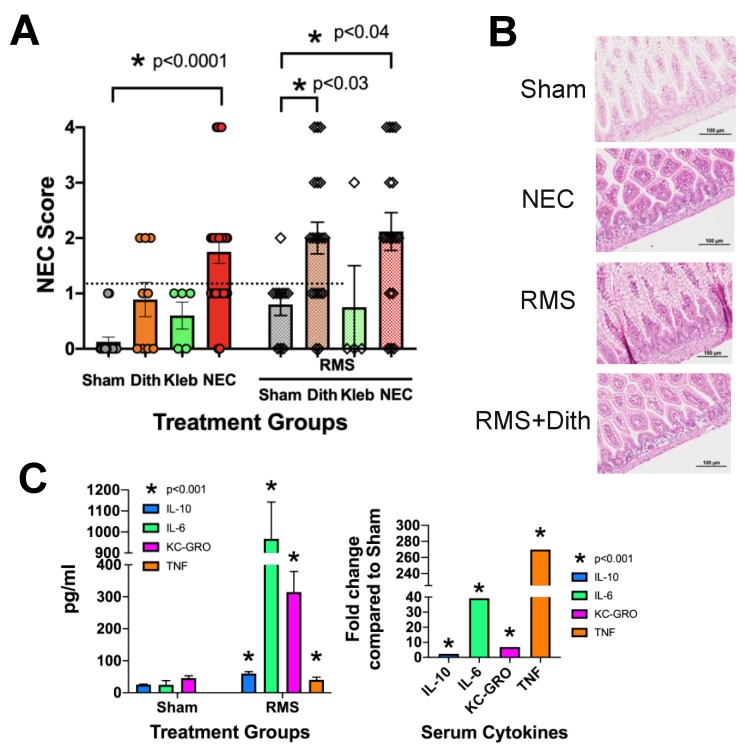
Formula feeding induces inflammation and removes the requirement for bacterial exposure to induce necrotizing enterocolitis (NEC)-like injury. C57Bl/6J mice (14–16 days old) were given an intraperitoneal injection of dithizone (75 mg/kg), a gavage feeding of Klebsiella pneumonia, the combination of both, or sham controls (*n* > 7 for all groups). Only the group given both treatments had significantly elevated injury scores. A second group of mice was given the same treatments but was also fed four times with 250 μL/feed of prepared rodent milk substitute (RMS). In this group, both the dithizone alone and the dithizone plus Klebsiella groups had significant injury, showing that adding RMS feeds eliminated the requirement for bacterial exposure (*n* > 7 for all groups, p values as shown) (**A**). Representative histology is shown (**B**). Serum samples were obtained from sham and sham-RMS mice and quantified for IL-10, IL-6, KC/GRO, and TNF. Sham-RMS mice had significantly higher serum levels of all cytokines than non-RMS control groups (*p* < 0.001 for all cytokines, *n* > 7 for all groups) (**C**).

Our previous studies have shown that induction of Paneth cell dysfunction followed by exposure to an enteral bacterial challenge induces NEC-like injury [22,24,54]; however, as opposed to several other common models of NEC [13,59,60], our model does not include formula feeding as a required component. To understand the effect of adding formula exposure to the Paneth cell-disruption model of NEC, we fed a separate group of mice 250 µL of prepared rodent milk substitute (RMS) [23,53,56] every three hours for four feeds prior to and during injury induction and compared them to controls. NEC-like injury was scored as previously described [21,22,23,24,31]. Interestingly, animals fed RMS no longer needed additional bacterial exposure to induce significant levels of intestinal injury (Figure 1A). Rather, exposure to enteral RMS feeds, in addition to induction of Paneth cell disruption alone, was enough to induce NEC-like injury equivalent to mice that were exposed to both Paneth cell disruption and *Klebsiella*-induced dysbiosis. Representative histology of all treatments can be seen in Figure 1B. To determine if RMS feeds were inducing inflammation, serum samples were collected at the end of the experiment and measured for Infγ, IL-10, IL-1β, IL-6, KC-GRO (murine homologue of IL-8), and TNF, which are cytokines often associated with intestinal injury and human NEC [61]. Exposure to RMS significantly increased serum levels of all cytokines (*n* > 7 for all groups, *p* < 0.001 for all cytokines measured) (Figure 1C).

### 3.2. Paneth Cell Disruption/Formula-Induced NEC is not Dithizone-Dependent

**Figure 2:**
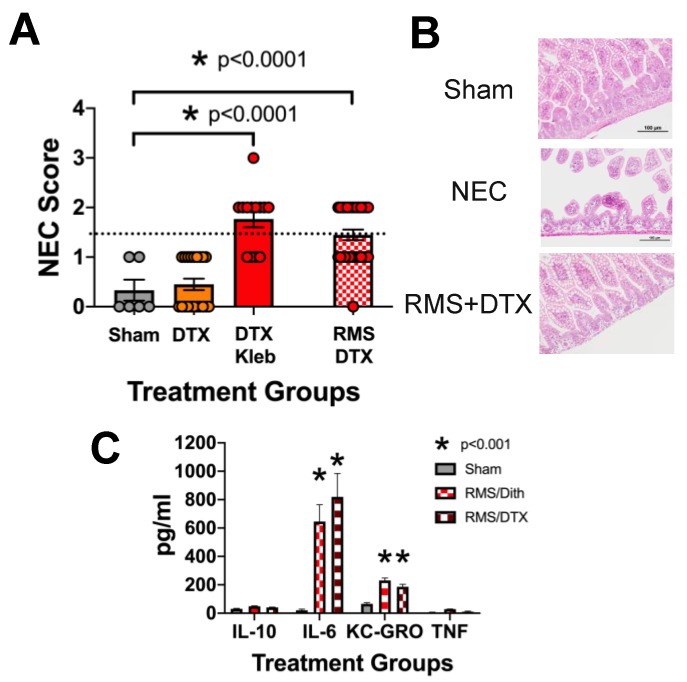
Formula feeding along with Paneth cell disruption resulting in NEC-like injury in the absence of bacterial dysbiosis is not dependent on the method of Paneth cell disruption. *PC-DTR* mice on a C57Bl/6J background (14–16 days old) were given an intraperitoneal injection of diphtheria toxin (40 ng/g), a gavage feeding of 1 × 10^8^/g Klebsiella pneumonia, the combination of both, or RMS in place of K. pneumoniae (*n* > 5 for all groups). Mice fed formula along with diphtheria toxin-induced Paneth cell disruption experienced significant NEC-like injury in the absence of Klebsiella-induced dysbiosis (**A**). Representative histology is shown (**B**). Serum samples obtained from mice before tissue harvest depict significant increases in IL-6 and KC-GRO compared to controls in both dithizone/RMS and DTX/RMS (*n* >10, *p* < 0.001) (**C**).

We next sought to determine if the method of Paneth cell disruption in combination with RMS impacted the level of injury and inflammation. P14–P16 *PC-DTR* mice were intraperitoneally treated with 25 ng/g body weight diphtheria toxin to induce Paneth cell apoptosis in addition to four 250 µL RMS feeds given via gavage every three hours for a total of four feeds. Similar to the dithizone/RMS model, diphtheria toxin/RMS treatment induced significant increases in intestinal injury without needing the addition of *Klebsiella*-induced dysbiosis (*n* > 5 for all groups, *p* < 0.0001) (Figure 2A). Representative histology of all treatments can be seen in Figure 2B. We additionally quantified the inflammatory cytokines present in the serum of the *PC-DTR* mice and found significant increases in IL-6 and KC-GRO (Figure 2C).

### 3.3. Immature Murine Generalized Intestinal Injury and Newborn Mortality Induced by Formula Feeding Is Osmolality-Dependent

**Figure 3:**
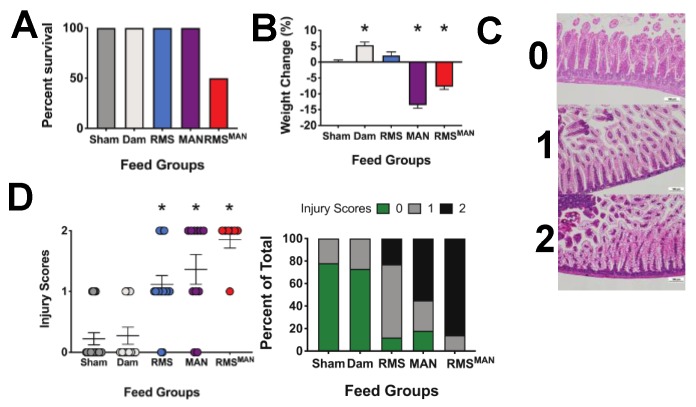
Exposure to increasing osmolality induces significant intestinal injury and mortality. C57BL/6J mice were given four 250 μL feeds with one of the following: sham Pedialyte (250 mOsm/kg H_2_O), dam feeds (300 mOsm/kg H_2_O), RMS (721 mOsm/kg H_2_O), MAN in saline (873 mOsm/kg H_2_O) or RMS + 10% mannitol (RMS^MAN^) (1491 mOsm/kg H_2_O). Mice fed RMS^MAN^ had a 50% mortality compared to all other groups (**A**). Mouse weight significantly increased in dam fed animals (5.4% increase, *p* = 0.009) compared to controls. Animals fed MAN exhibited a significant loss of weight (−13.6%, *p* < 0.0001), as did animals fed RMS^MAN^ (−7.8%, *p* < 0.0001) (**B**). Intestinal injury was determined on a three point injury scale as shown in (**C**). Intestinal injury scores were significantly higher in the RMS, MAN, and RMS^MAN^ groups compared to sham controls (*p* = 0.0023, 0.0009, and < 0.0001 respectively) (**D**).

RMS has an osmolality of 721 mOsm/kg H_2_O, which is more than twice as high as either Pedialyte (250 mOsm/kg H_2_O), saline (273 mOsm/kg H_2_O), or most mammalian milk (300 mOsm/kg H_2_O) [44,53]. To determine if isolated osmolality was responsible for the changes we observed in our NEC model, we fed mice every three hours for a total of four feeds with one of the following: saline (273 mOsm/kg H_2_O), ad libitum dam feeding (300 mOsm/kg H_2_O), RMS (721 mOsm/kg H_2_O), 10% MAN in saline (873 mOsm/kg H_2_O), or RMS + 10% MAN (RMS^MAN^) (1491 mOsm/kg H_2_O). Importantly, animals fed the highest osmolality formula (RMS^MAN^) had a 50% mortality (most of them prior to the fourth feed) compared to 100% survival for all other feed types (Figure 3A). As mannitol can act as a significant diuretic, we also monitored weights of the animals at the beginning and end of experimentation (Figure 3B). While dam fed animals experienced a significant increase in weight (5.4% increase, *p* = 0.009), sham animals had negligible weight change. Animals fed RMS alone had a slight but non-significant weight increase (2.1%, *p* = 0.27). Animals fed MAN exhibited a significant loss of weight (−13.6%, *p* < 0.0001), as did animals fed RMS^MAN^ (−7.8%, *p* < 0.0001). To quantify generalized intestinal injury, distal ileal sections were harvested at time of euthanasia and scored by two separate blinded investigators on a three-point scale of small intestinal injury described previously [55,58] that is distinct from the NEC injury scoring system (Figure 3C). Injury scores were significantly higher in the RMS, MAN, and RMS^MAN^ groups compared to sham controls (*p* = 0.0023, 0.0009, and < 0.0001 respectively) (Figure 3D).

**Figure 4:**
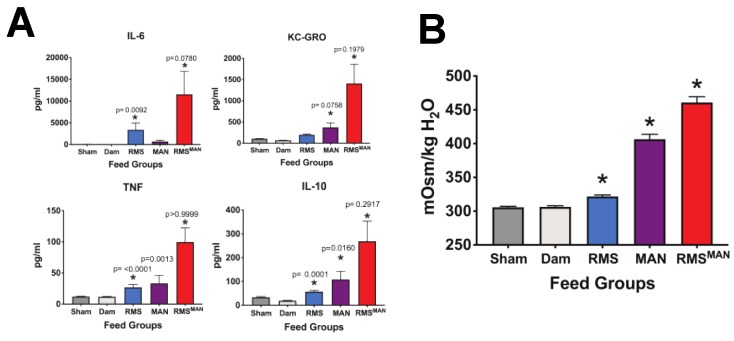
Exposure to increasing osmolality induces significant increases in serum inflammation. Serum samples were obtained at the time of euthanasia and quantified for the presence of IL-6, KC-GRO, TNF, and IL-10 (A). No significant differences were seen between sham and dam feeds. RMS feeds significantly induced IL-6, IL-10, and TNF levels. MAN feeds significantly increased serum levels of KC-GRO and IL-10, while RMS^MAN^ feeds significantly increased serum levels of all cytokines evaluated (*n* > 5 for all groups, p values as shown) (**A**). Serum osmolality was also quantified to ascertain if enteral feeds were impacting the animal systemically. Feeds with MAN, RMS, or RMS^MAN^ all significantly increased serum osmolality, although the highest elevations were in feeds containing mannitol (*n* > 5, *p* < 0.0001 for all comparisons) (**B**).

Next, we wanted to assess the impact of these different osmolality feeds on serum inflammation (Figure 4A). Serum samples were obtained at the time of euthanasia and quantified for the presence of IL-6, IL-10, TNF, and KC-GRO. Sham and dam feeds showed similar levels of all four cytokines. RMS feeds significantly increased IL-6, TNF, and IL-10 levels compared to sham controls. Exposure to MAN feeds caused significant serum elevations of KC-GRO and IL-10, while feeding with the high osmolality RMS^MAN^ induced significant elevations in all four cytokines (n > 5 in all groups and *p* < 0.05 with specific *p* values shown). Lastly, we examined serum osmolality to ascertain if enteral feeds were impacting the animal systemically (Figure 4B). Serum was drawn from animals at time of euthanasia and measured for osmolality. Feeds with RMS, MAN, or RMS^MAN^ all induced statistically significant elevations in serum osmolality, although the highest elevations were in feeds containing mannitol (n > 5, *p* < 0.0001 for all comparisons).

### 3.4. Exposure to Mannitol-Increased Osmolality Induced Significant Alterations in the Composition of the Cecal Microbiome in the Immature Intestine 

**Figure 5:**
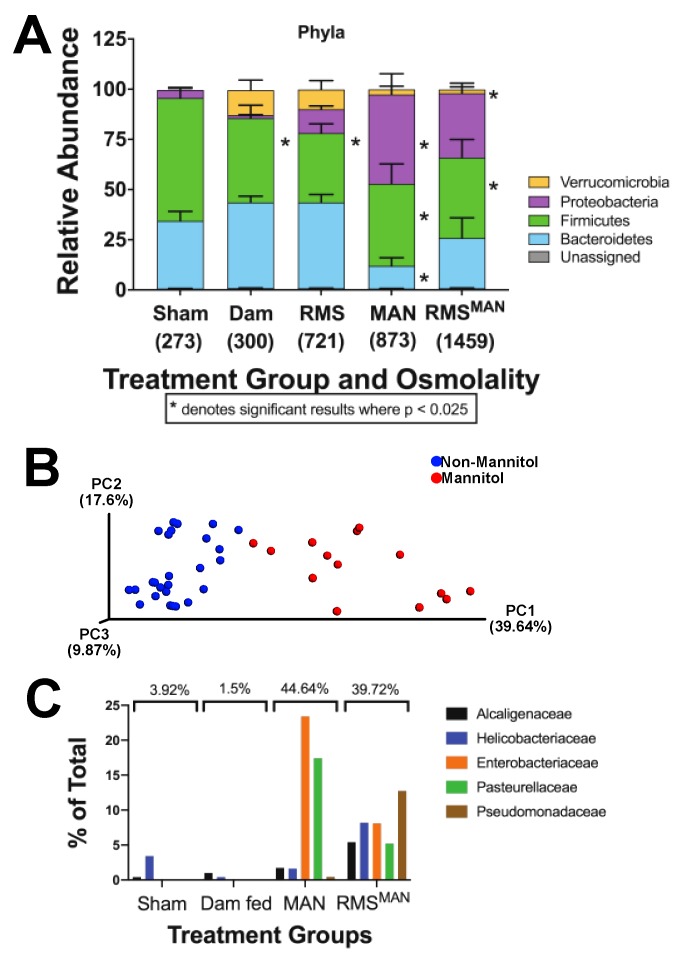
Exposure to increased osmolality induced significant alterations in the composition of the cecal microbiome in the immature intestine. Feed substance induced alterations in the microbial composition of the cecum. Dam fed and RMS fed mice had significantly less Firmicutes than sham controls, while mice fed MAN and RMS^MAN^ had a shift in the microbiome resulting in a significant increase in Proteobacteria composition (*n* = 9 animals per group, * represents a significant change from sham controls where *p* < 0.025) (**A**). Principal coordinate analysis revealed that animals fed mannitol had distinct clustering of their flora compared to all other groups without mannitol in their feeds (**B**). Within the Proteobacteria phylum, the MAN condition resulted in increases in Enterobacteriaceae and Pasteurellaceae compared to controls (**C**). In the RMS^MAN^ condition, increases were seen, compared to controls, to approximately the same amount in Alcaligenaceae, Helicobacteraceae, Enterobacteriaceae, Pasteurellaceae, and Pseudomonadaceae (**C**).

One possible mechanism responsible for the osmolality-induced injury and inflammation was through alteration of the intestinal microbiome. To analyze this, cecal samples were collected following euthanasia and quantified for microbial composition. All mice receiving feeds experienced shifts in their cecal bacterial flora compared to those receiving only saline (Figure 5A). Mice fed RMS alone had a significant decrease in Firmicutes species similar to what was seen in dam fed mice. Interestingly, there were no differences in the microbiome between mice fed formula and mice that were dam fed. Mice fed either MAN or RMS^MAN^ had significant increases in Proteobacteria with compensatory decreases in the relative number of Bacteroidetes and Firmicutes species. Principle coordinate analysis revealed that animals fed diets containing mannitol (MAN, RMS^MAN^) had microbiome compositions that were discrete from those fed diets not containing mannitol (Sham, Dam, RMS) as depicted by the distinct clustering of the samples (Figure 5B). Within the Proteobacteria phylum, the families with the biggest increase in the MAN condition compared to sham or dam fed was Enterobacteriaceae and Pasteurellaceae (Figure 5C). Interestingly, in the RMS^MAN^ condition trending increases were seen compared to sham or dam fed to approximately the same amount in Alcaligenaceae, Helicobacteraceae, Enterobacteriaceae, Pasteurellaceae, and Pseudomonadaceae (Figure 5C).

### 3.5. Osmolality-Induced Effects Are Dependent on the Methodology Used to Increase the Solute Level

To determine if increases in injury, inflammation, and bacterial dysbiosis were dependent on MAN or high osmolality, we created a formulation of RMS using three times the salt concentration in place of mannitol to induce a second method for obtaining a high osmolality (RMS^SALT^) (982 mOsm/kg H_2_O) feed. Experiments were performed as above using triple salt (SALT) in place of MAN.

**Figure 6:**
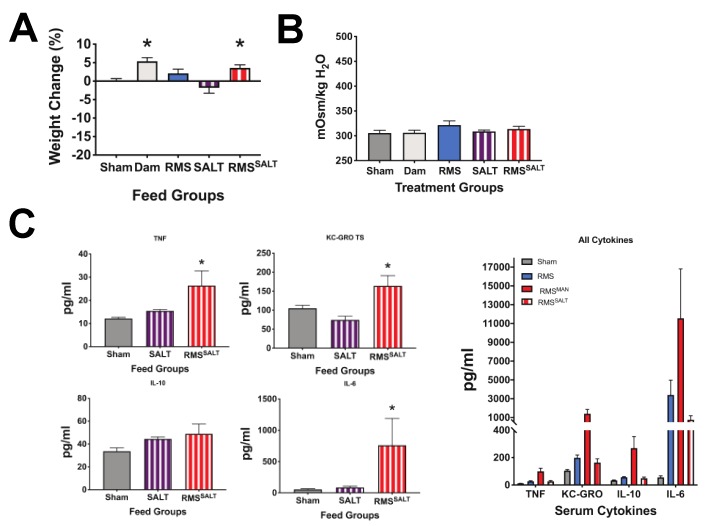
Osmolality-induced effects are methodology-dependent. To determine if our results were due to osmolality or to MAN, an additional high osmolality feed was generated by adding three times the salt concentration to standard RMS (RMS^SALT^). Feeds with SALT and RMS^SALT^ did not show the significant weight loss seen with MAN feeds (**A**), and no change in serum osmolality was observed in SALT or RMS^SALT^ fed groups when compared to controls (**B**). SALT feeds had no significant impact on serum cytokine levels, while RMS^SALT^ feeds induced significant increases in IL-6, KC-GRO, and TNF compared to sham controls (*n* > 4 for all experiments, *p* = 0.0019, 0.0092, and 0.0003 respectively) (**C**). The far-right panel shows the combined MAN and SALT data for more direct comparison of the degree of inflammation generated by each feed type.

**Figure 7:**
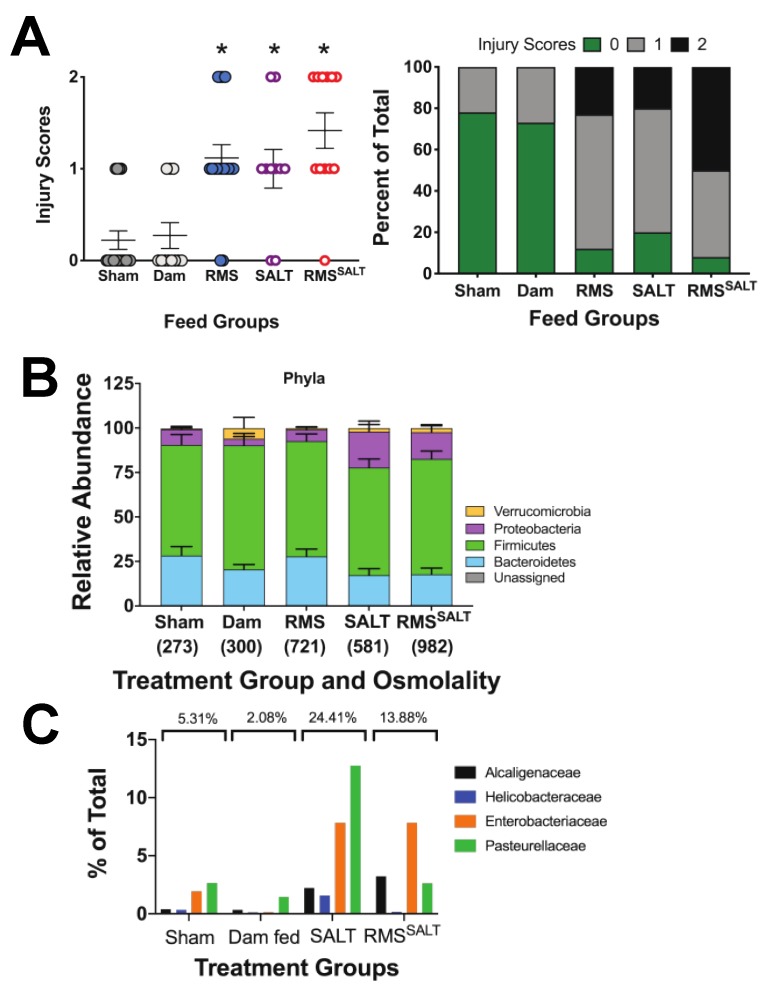
Alteration of the microbial composition is methodology-dependent, but intestinal injury is methodology-independent and is related instead to the height of osmolality achieved. Similar to our high osmolality MAN groups, feeding with either SALT alone (581 mOsm/kg H_2_O) or RMS^SALT^ (982 mOsm/kg H_2_O) had significantly increased generalized injury scores compared to controls (*n* = 7, *p* = 0.0196 and < 0.0001 respectively) (**A**). However, no significant changes in microbial phylum composition were seen when comparing SALT and RMS^SALT^ feeds to controls (**B**). When comparing changes in the Proteobacteria families similar to the comparison made in the mannitol conditions, trending increases were seen in both Enterobacteriaceae and Pasteurellaceae in the SALT and RMS^SALT^ conditions compared to sham- and dam fed mice (**C**).

As opposed to feeding with MAN, SALT feeds induced no mortality (data not shown as all animals survived), nor significant weight loss (Figure 6A). Further, no change in serum osmolality was seen from controls (Figure 6B). In looking at effects of SALT feeds on serum inflammation, feeding with SALT alone (equivalent salt concentration dissolved in saline) (581 mOsm/kg H_2_O) had no significant effects on TNF, KC-GRO, IL-10, or IL-6 compared to controls, while feeding with RMS^SALT^ (982 mOsm/kg H_2_O) significantly increased serum TNF, KC-GRO, and IL-6 levels (Figure 6C). Importantly, while RMS^SALT^ feeds induced cytokine elevation, they were markedly less than what was seen from RMS^MAN^ feeds (Figure 6C—data combined from 4A and 6C for ease of comparison). In quantifying intestinal injury, feeds with both SALT and RMS^SALT^ had significantly increased injury scores compared to sham controls and were similar to those seen with RMS alone (Figure 7A). As was seen in our MAN experiments, the feeds with the highest osmolality (RMS^SALT^) had the greatest percentage of maximal injury scores, however, as was seen in our cytokine levels, feeds with RMS^SALT^ did not reach the same degree of injury as that seen in RMS^MAN^. Interestingly, the microbiome showed no significant differences in any phyla when treatment groups were compared to the dam or to sham fed mice (Figure 7B). Although we did not see any shifts at the phylum level, when looking at changes in Proteobacteria families we did see trending increases in both Enterobacteriaceae and Pasteurellaceae in the SALT and RMS^SALT^ conditions compared to sham- and dam fed mice (Figure 7C).

### 3.6. High Osmolality RMS Deceased Survival When Included in Dithizone Paneth Cell Disruption and Formula Feeding NEC Model

**Figure 8:**
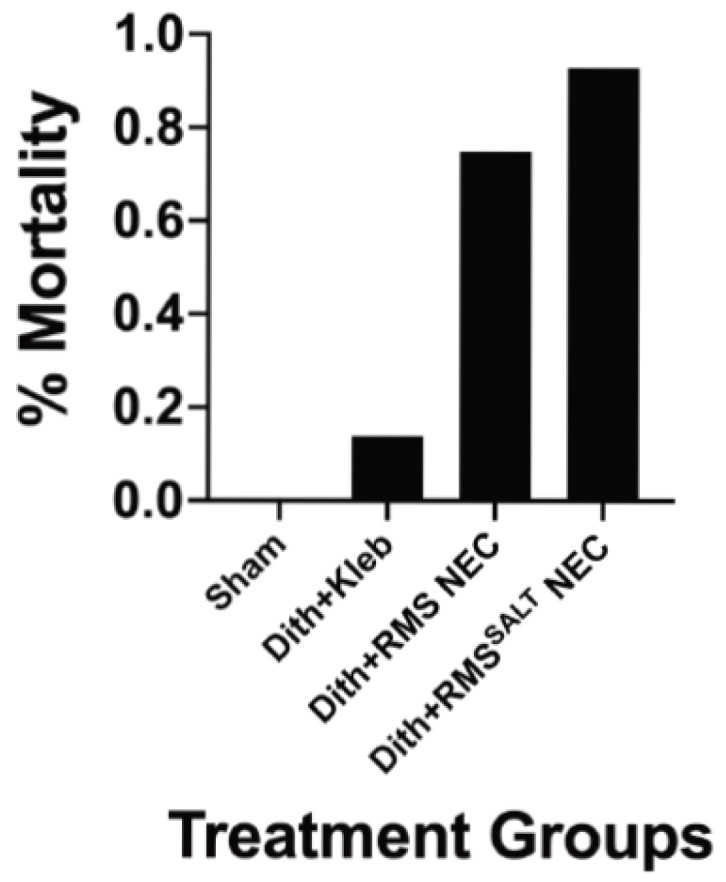
High osmolality formula combined with Paneth cell disruption results in increased mortality compared to regular formula. C57Bl/6J were injected with dithizone or an equivalent volume of LiCO_3_ (controls animals) and exposed to feeds with Pedialyte, 1 × 10^11^/kg *Klebsiella pneumonia*, regular RMS, or RMS^SALT^ (*n* = 11 Sham, 36 Dith+Kleb, 28 Dith+RMS, 28 Dith+RMS^SALT^). Animals exposed to RMS^SALT^ had increased mortality (93%) compared to those fed regular RMS (75%) or gavage of *K. pneumonia* (14%).

Our data shows that formula feeding along with Paneth cell disruption results in significant increases in inflammatory cytokines and in NEC-like injury. In addition, our data shows that exposure of the immature intestine to high osmolality formula (without Paneth cell disruption) significantly increases generalized intestinal injury and serum inflammation when compared to feeds with lower osmolality. Based on this data, we wanted to study the impact of high osmolality formula in one of our newly described RMS NEC models to determine the impact on survival. We disrupted Paneth cells using dithizone and gavage fed 250 µL of RMS with or without high salt to increase the osmolality (as seen in Figure 1 and Figure 2). We found that in these conditions, mice that were fed high osmolality formula had higher mortality rates (93% mortality) compared to their regular RMS fed counterparts (75% mortality) (Figure 8).

## 4. Discussion

The goal of this study was to determine the impact of formula feeding in combination with Paneth cell disruption and dysbiosis on rates and severity of injury to the immature intestine. Our secondary goals were to determine if high osmolality feeds would impact the immature intestine’s susceptibility to injury, inflammation, or alterations in the microbiome composition, and to determine if high osmolality would exacerbate mortality in a formula NEC model. Our desire was to address gaps in knowledge regarding the mechanisms behind the increased rates of NEC in infants fed formula as well as questions regarding inconsistencies in data about the role of high osmolality and subsequent susceptibility to develop NEC. Our initial hypothesis was that formula feeding would exacerbate NEC-like injury in the Paneth cell-disruption NEC model. Interestingly, we found that formula feeding along with dithizone or diphtheria toxin to induce Paneth cell disruption caused NEC-like injury in our mice while eliminating the requirement of inducing a secondary bacterial dysbiosis. We further hypothesized that increased osmolality would lead to increased intestinal injury and inflammation through an alteration of the intestinal microbiome. Our novel data show that mice fed high osmolar feeds experience increased serum inflammation and increased levels of intestinal injury compared to sham controls. However, we found that these effects were independent from alterations in the microbiome. Finally, we hypothesized that high osmolar formula would exacerbate mortality seen in our formula NEC model and this was supported by our data. These data are critical to NEC biology and the field of neonatology as the imperative task of providing adequate nutrition to preterm infants requires the fortification of feeds to achieve the caloric requirements to maintain the fetal growth trajectory. Our data adds important insight into the role that formula feeds, and specifically high osmolar formula feeds, have on susceptibility to injury of the immature intestine.

The field of neonatology has advanced tremendously over the past few decades, yet despite these advances there has been slow progress on elucidating the pathophysiology behind NEC. While human breast milk is protective against NEC [17,35], it remains unknown why. The suggestion that hyperosmolality of prepared formulas could induce NEC was first postulated in the 1970s. In 1975, Santulli et al. published a case series reviewing 64 infants with NEC born from 1955 through June 1974. As part of the conclusion of this seminal work, Santulli suggested that hyperosmolar feeds may be causative of NEC as some of the infants who developed NEC had received hyperosmolar feedings (750 mOsm/L) [44,46]. That same year, Book et al. published a prospective study of 16 infants weighing less than 1200 g who were randomized to receive either elemental formula or cow milk formula [45]. Seven of eight (87.5%) infants fed the elemental formula (650 mOsm/L) and two of eight (25%) fed the standard cow milk formula (359 mOsm/L) developed necrotizing enterocolitis (*p* < 0.02). Based on these data, the authors concluded that the hypertonicity of the elemental diet may have contributed to the increased incidence of NEC in infants fed this formula. Following these two manuscripts, the American Academy of Pediatrics (AAP) in 1976 developed a recommendation that the osmolarity of infant formula should be less than 400 mOsm/L [47]. As osmolarity is defined as the number of solute particles in 1L of solvent and osmolality is the number of solute particles in 1kg of solvent, the AAP recommendations approximates to an osmolality of 450 mOsm/kg H_2_O [62]. This was a consensus view based on the observation that the milk of most mammalian species has an osmolarity of approximately 300 mOsm/L and that hyperosmolar formulas may be a causative factor in NEC. Despite (or perhaps because of) this consensus statement, little data has been generated to support the theory that osmolality is detrimental. Recent works by the Pierro lab have begun to address this gap in understanding. Their work utilizes five- to nine-day-old mice who were given systemic hypoxia over several days as well as hyperosmolar feeds laced with LPS three times a day. In their studies, the Pierro lab has shown that formula feeding induces changes in intestinal microcirculation and that the associated injury is not osmolality-dependent [48,49]. However, our data differs in that our mice were significantly older from a developmental pattern standpoint [20] and our studies used a much broader range of osmolalities as well as two different methods of altering feed osmolality. In this way, our data expands and adds to the important previous work of the Pierro group.

Although current neonatal practice is to use low osmolality feeds in the neonatal intensive care unit (NICU), these formulas are frequently fortified with supplemental protein, as well as other substances essential for growth such as vitamins or minerals to provide adequate nutrition. It is important to realize that use of the fortifiers and other supplements greatly increase osmolality. Our data show that feeding just four feeds of high osmolar formula to neonatal mice can provoke significant increases in serum inflammation and in epithelial damage in the immature small intestine. This appears to be dose-dependent, as feedings with higher osmolality had higher inflammation and injury scores. While breast milk, amniotic fluid, and Pedialyte all have osmolalities below 350 mOsm/kg H_2_O, our formulas were significantly higher: SALT (581 mOsm/kg H_2_O); RMS (721 mOsm/kg H_2_O); MAN (873 mOsm/kg H_2_O); RMS^SALT^ (982 mOsm/kg H_2_O); and RMS^MAN^ (1491 mOsm/kg H_2_O). Importantly, our injury scores for RMS^SALT^ and MAN alone (982 and 873 mOsm/kg H_2_O, respectively) were similar, as were the injury scores for RMS alone and SALT (721 and 581 mOsm/kg H_2_O, respectively). The highest generalized injury scores were observed with the highest osmolality formula, RMS^MAN^ (1491 mOsm/kg H_2_O). While the osmolality of acceptable NICU feeds is below the levels that induced injury in our model, the formula and standard fortifiers commonly used do not take into consideration other substances that are often used with feeds in the NICU. A recent paper by Chandran et al. examined fourteen common medications utilized in the NICU [63]. Of these medications, nine had osmolalities too high to measure and the five additional medications were all above 450 mOsm/kg H_2_O. This list included iron, multi-vitamins, NaCl, NaPO_4_, and KPO_4_, which are all common additives to feeds. To keep the osmolality below 450 mOsm/kg H_2_O, the medications required significant dilutions of up to 1:40 when mixing the medication in preterm formulas and 1:250 when mixing with fortified expressed breast milk [63].

We observed a trend of increased injury being associated with higher osmolality feeds, but interestingly this trend was independent of the method for generating higher osmolality. While we were concerned that MAN would cause injury through dehydration, use of high salt formula did not impact weight while still inducing intestinal damage. This contrasts the work by Miyake et al. [48]. In this work, there were no differences seen in injury generation between formulas with an osmolality of 849 and 325 mOsm/kg H_2_O. However, all animals also underwent exposure to LPS four mg/kg/day and hypoxia three times a day for 10-min time periods to induce NEC-like injury; thus, it is possible that the injury sustained in the hypoxia-formula model of NEC is severe enough that it would overwhelm any injury patterns seen in high osmolality alone. Although there is still conflicting data on the effect of formula osmolality and its relation to NEC likely due to conditional differences, our data highlights the danger of high osmolality not just in causing intestinal injury and inflammation, but also mortality as well when high osmolality formula is combined with Paneth cell disruption. Interestingly, our data suggested that the injury, inflammation, and mortality observed was related to the osmolality itself and could occur without changes in the microbiome, suggesting, at least for the microbiome, what is used to increase the osmolality is more indicative of community type than the osmolality itself. Additionally, it suggests that intestinal injury and inflammatory cytokine levels are likely independent of the microbiome in this particular model as intestinal injury and inflammation can be increased without shifts in the microbial community.

These findings were in direct contrast to our original hypothesis as we predicted increased osmolality would lead to increased intestinal injury and inflammation through an alteration of the intestinal microbiome. We were not surprised to find that different feed formulations had differential effects on the microbiome, but we were surprised to find that they were independent of injury scores. One finding was that feeds containing mannitol had significant expansions of the Proteobacteria phyla and more specifically Enterobacteriaceae. This is interesting as many studies have associated similar blooms with development of NEC [10,64]. Furthermore, many studies have linked formula feeding with NEC, and one connection made is that formula feeding tends to result in differential colonization of the infant gut compared to breast feeding. While breast fed infants tend to have a predominance of Firmicutes and Actinobacteria, formula-fed infants tend to have a predominance of Proteobacteria and more specifically Enterobacteriaceae, which can put them at higher risk for bacterial dysbiosis, which is another risk factor for NEC [65]. Another interesting finding was that in salt conditions we did not observe significant changes in the microbiome at the phylum level, but when looking at family level changes, we saw a trending increase in Enterobacteriaceae in the higher osmolality SALT and RMS^SALT^ conditions. These conditions were associated with higher generalized injury scores; however, injury scores and inflammation were more consistent on overall osmolality than alterations in the cecal microbiome. One possibility for the increase in specific groups of organisms with mannitol feeds is that many Proteobacteria and Firmicutes species can utilize mannitol as an energy source allowing for a selective expansion of the bacteria that can utilize mannitol, which was supported by our data [66,67,68]. Additionally, Proteobacteria and Firmicutes species tend to be more tolerant to high salt conditions compared to other phyla of bacteria, which may explain why there were trending increases in some Proteobacteria families [69,70]. These data suggest that although changes in the microbiome were present in both the mannitol and salt related conditions, they likely occurred independent of the inflammation and injury we observed and were not causative in these experiments.

Although we did not observe microbiome alterations between dam fed and formula-fed mice, we did find significant increases in inflammation in formula-fed animals compared to dam fed animals. Formula feeding has been linked with increases in inflammation, which may explain why it can replace bacterial dysbiosis in our model and serve as a secondary hit in other NEC models [18]. Similar to other labs that have shown that formula feeding along with some other secondary form of insult such as hypoxia/hypothermia, LPS, or bacterial insult can induce NEC, we saw that when we induced Paneth cell disruption using either dithizone or diphtheria toxin and followed this with formula feeding of RMS we saw significant increases in NEC-like injury [13,15,18,48,59]. Unsurprisingly, the injury we observed was slightly different based on the type of Paneth cell disruption induced. Slightly higher NEC scores were found in the dithizone RMS NEC model and the damage in the intestine was mainly confined to the base and the core of the villi. While the scores were still significant in the diphtheria toxin RMS NEC mice, the scores trended to be slightly lower on average compared to the dithizone RMS NEC. Additionally, the damage pattern in the intestine included both the base of the villus as well as vacuolized cells at the top of the villus down compared to the damage pattern in the dithizone RMS NEC. This supports the findings of Lueschow et al. in 2018 showing that the NEC scores with diphtheria toxin and bacterial gavage, although still significant, trended slightly lower on average compared to the scores seen with dithizone and bacterial gavage [31]. Additionally, the difference in damage patterns of the intestine between dithizone and diphtheria toxin that we observed while formula feeding is complemented by the two different types of Paneth cell disruption described in Lueschow et al., 2018 where dithizone induces upregulation of autophagy pathways while diphtheria toxin induces apoptosis [31,71]. The findings that formula can replace induction of bacterial dysbiosis in a Paneth cell disruption NEC model highlights the potential dangers of formula feeding in premature infants at heightened risk for NEC.

Ultimately, the results of this paper demonstrate that formula feeding can induce NEC-like injury when combined with Paneth cell disruption without induction of bacterial dysbiosis, which is likely due to the inflammatory nature of the RMS formula without any additives. Additionally, high osmolality formula without Paneth cell disruption results in increased generalized intestinal injury and inflammation to a greater degree than formula feeding on its own in a microbiome independent manner. When high osmolality formula was combined with Paneth cell disruption, higher mortality rates were seen compared to when RMS alone was combined with Paneth cell disruption. Furthermore, the results show that changes in the microbiome can occur independent of injury and inflammation and were not causative in this case, as injury and inflammation were more closely connected with osmolality than changes in the microbiome. This is a critical point to consider as osmolality, microbiome composition, and intestinal injury can be interrelated or completely independent of each other depending on the conditions present. Therefore, as we consider optimized feeds that maximize nutrition for preterm infants, we must be vigilant of what we are giving to make sure we protect their immature intestinal tract.

## Figures and Tables

**Table 1:** Definitions of the abbreviations for the feeds used in this paper along with the associated osmolality of each feed type.

Feeding Type/Abbreviation	Formulation	Osmolality (mOsm/kg H_2_O)
Control	Saline	250
RMS	Rodent milk substitute	721
Dam	Ad libitum dam feeding	300
Saline	Saline	273
MAN	10% Mannitol in saline	873
SALT	Saline + 0.7 g NaCl	581
RMS_MAN_	RMS + 10% mannitol	1491
RMS_SALT_	RMS + 0.7 g NaCl	982
